# Continuous Hypodynamic Change of Cerebrospinal Fluid Flow as A Potential Factor Working for Experimental Scoliotic Formation

**DOI:** 10.1038/s41598-020-63822-x

**Published:** 2020-04-22

**Authors:** Zhi Zhao, Tao Li, Ni Bi, Zhiyue Shi, Ying Zhang, Quan Li, Yingsong Wang, Jingming Xie

**Affiliations:** Department of Orthopaedics, The 2nd Affiliated Hospital of Kunming Medical University, Kunming, 6500101 Yunnan People’s Republic of China

**Keywords:** Experimental models of disease, Neuromuscular disease, Pathogenesis

## Abstract

Scoliosis is often associated with syringomyelia (SM). As an important role in SM formation, the influence from abnormal cerebrospinal fluid (CSF) flow is still unclear to scoliosis. The aim of this experimental work is to explore the connection between CSF flow and scoliosis through imaging and histological analysis on the basis of a kaolin-induced scoliotic rabbit model. For imaging observation, in 40 kaolin-induced rabbits by C7 spinal cord injection, through pre- and postoperative MRI and radiography, CSF flow and scoliosis formation were detected at consecutive phases. According to the final formation of scoliosis until postoperative week 12, the kaolin-induced rabbits were divided into 2 groups. Through comparing the 2 groups, the relationship between the changes of CSF flow velocity and scoliosis formation were reviewed and analyzed. For histological observation, another 20 kaolin-induced rabbits were used for consecutive histological observations of spinal cord at postoperative 3-day, 2-week, 4-week and 6-week. After kaolin-induction, abnormal spinal coronal curve was observed from postoperative week 6 in the 37 survived rabbits. At postoperative week 12, scoliosis formation was detected in 73.0% kaolin-induced rabbits and the mean Cobb angle was 27.4°. From the comparison between scoliotic and non-scoliotic groups, the difference of the velocities of CSF flow was more obviously from postoperative week 4 to 12, especially after week 6. In the scoliotic group, the peak velocity of CSF flow was diseased gradually following scoliosis formation after induction. Moreover, the decrease of the peak velocities of CSF flow from preoperation to postoperative 12 weeks (ΔVmax), including up-flow (ΔVUmax) and down-flow (ΔVDmax), were positively correlated to the final scoliotic Cobb angle (*P* < 0.01). Through histological observation at different phases, the distinctive pathological changes of the spinal cord included early inflammatory reaction, adhesion and blockage in the subarachnoid space and the central canal, perivascular space enlargement, central canal expansion, which suggested the CSF flow being blocked by multiple ways after kaolin-induction. In conclusion, experimental scoliosis can be successfully induced by intraspinal kaolin injection. In this model, continuous hypodynamic change of CSF flow was correlated to the formation of scoliosis, which could be an important factor of scoliotic pathogenesis being explored furtherly.

## Introduction

Scoliosis is often associated with syringomyelia (SM) in clinical, which can be found in 25–85% of the patients being diagnosed as SM^1^. Meanwhile, SM also can be detected by magnetic resonance imaging (MRI) in 19.5% infantile, 26% juvenile and 20% adolescent patients ever being diagnosed as idiopathic scoliosis (IS)^2^. Moreover, in the patients with severe scoliosis (Cobb angle more than 90°), the rate of associated-SM is even up to 60.4%^3^. However, the relationship between scoliosis and SM is still obscure, which has been explained as a causal link through the “paraspinal muscle denervation” theory: the pressure from an asymmetrically expanded syrinx may cause the disturbances in innervation of the trunk musculature and thus causing the imbalance that leads to resultant scoliosis^4–7^. However, this hypothesis could not explain several clinical phenomena reasonably, such as the inconsistence between the magnitude of scoliosis and the size of syrinx, the direction of curve and the eccentricity of syrinx in the spinal cord, the site of syrinx and the apex of scoliosis, etc.^8–10^. Additionally, primary neurosurgical decompression can reduce syrinx and improve neurological function, but could fail to stabilize the associated scoliosis^11–13^. Therefore, the relationship between scoliosis and SM should not be believed as a simple causal link.

SM is a pathological state of the spinal cord as a result of trauma, malformation etc.^14–16^. As a crucial reason of SM, the hypodynamic change of cerebrospinal fluid (CSF) flow has been studied furtherly^17–20^. In the pathological process of SM-associated scoliosis, CSF flow hypodynamic change plays an important role in SM formation, but makes unclear effects to associated scoliosis.

Based on that consideration, in this study, on the basis of a rabbit scoliotic model being induced by injecting kaolin into spinal cord at the level of seventh cervical vertebra (C7), the following hydrodynamic changes of CSF flow in the process of coronal curve development and formation were recorded and analyzed consecutively by Phase Contrast–Cine MRI (PC-cine MRI) scanning. According to the final formation of scoliosis until postoperative week 12, the kaolin-induced rabbits were divided into scoliosis (group S) and non-scoliosis (group Non-S). Then in order to further understand the role of CSF flow in scoliosis formation, through comparison between the 2 groups, the relationship between the changes of CSF flow velocity and scoliosis formation were analyzed. Additionally, combined with histological observation at several consecutive phases after kaolin-induction, the postoperative extramedullary and intramendullary pathological changes of the spinal cord were observed for understanding how the modeling method to affect the CSF flow.

## Results

### Imaging detection results

In the 40 kaolin-inducted rabbits for imaging detections, 37 survived more than 12 weeks after operation and were included in the final experimental analysis. In the 3 died rabbits: 1 was related to the surgical process and possibly from mass blood loss, 2 was from the postanesthetic hypothermia. None occurred incision infection. Most kaolin-induced rabbits exhibited signs of lethargy and anorexia. None was dead in the shame animals.

### Scoliosis formation and grouping

**Figure 1:**
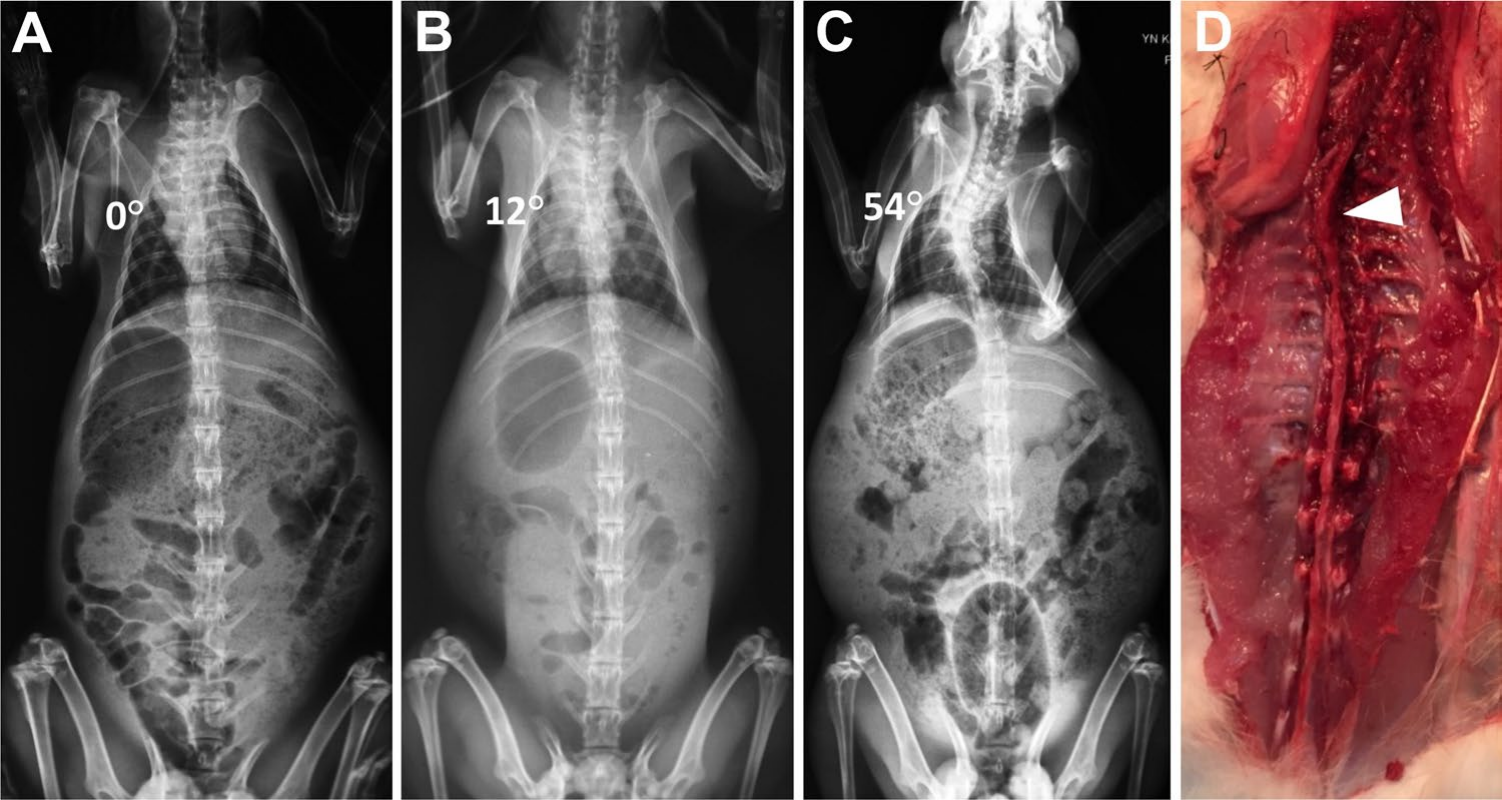
Posterior-anterior radiographs of the spine and scoliosis development (*Rabbit 21#*). Preoperative coronal imaging of spine (**A**). Postoperative 6-week, a tiny thoracic curve appearance and the Cobb angle was 12° (**B**). Postoperative 12-week, the thoracic scoliosis developed to 54° (**C**). Post-anatomic observation of the thoracic scoliosis and the apical vertebra was marked by the white arrow (**D**).

In the 37 survived kaolin-induced rabbits, before the week 6 after surgery, even minor curve of spine was found in none. But from the week 6 to 12, scoliosis formation was detected in 73.0% (27/37) of the experimental rabbits and the final Cobb angle was up to 27.4 ± 10.0° (15–59°) (Fig. 1, Table 1). These 27 rabbits were involved in group S, and the other 10 without spinal curve were in group Non-S. In the control, scoliosis formation was observed in none.

**Table 1:** The CSF Flow and Scoliosis Changes at Consecutive Phases in Group S.

			Pre-op	Post-op				
				Week 4	Week 6	Week 8	Week 10	Week 12
CSF Flow (cm/sec)	VDmax	C0	7.0 ± 1.6	5.7 ± 1.3	4.5 ± 1.1	4.1 ± 1.0	3.9 ± 1.0	3.9 ± 1.0
		C7	7.4 ± 1.8	6.0 ± 1.5	4.9 ± 1.2	4.4 ± 1.1	4.2 ± 1.0	4.2 ± 1.0
	VUmax	C0	7.6 ± 1.5	6.5 ± 1.6	5.3 ± 1.3	4.8 ± 1.3	4.5 ± 1.2	4.4 ± 1.2
		C7	8.1 ± 1.6	6.5 ± 1.3	5.4 ± 1.1	4.9 ± 1.0	4.6 ± 1.0	4.6 ± 0.9
Cobb angle (°)			0	0	3.1 ± 4.1	10.9 ± 9.2	19.3 ± 10.3	27.4 ± 10.0

*CSF indicates cerebrospinal fluid; VDmax, peak velocity of CSF up-flow; VUmax, peak velocity of CSF down-flow

**Figure 2:**
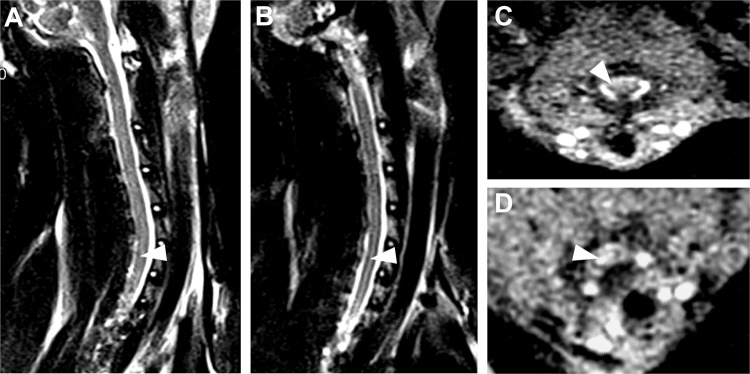
Pre- and postoperative 12-week MRI scanning of the cervical spine cord with kaolin-induction (*Rabbit 21#*). Preoperative sagittal and transverse MRI imaging of the cervical spine cord (**A,C**). Postoperative 12-week, the syrinx was observed in the sagittal and transverse MRI imaging of cervical spine cord (**B,D**).

Meanwhile, syrinx also started to be observed at postoperative week 6, and MRI scanning showed syrinx occurrence mainly in the cervical-thoracic segments. Syrinx was found in 70.3% (26/37) of the experimental rabbits at postoperative week 12 (Fig. 2). In groups S and Non-S, 74.1% (20/27) and 70.0% (7/10) of the animals were associated with syrinx separately (*P* > 0.05).

### The trends of CSF flow changes and scoliosis formation

#### Group S vs Group Non-S

**Figure 3:**
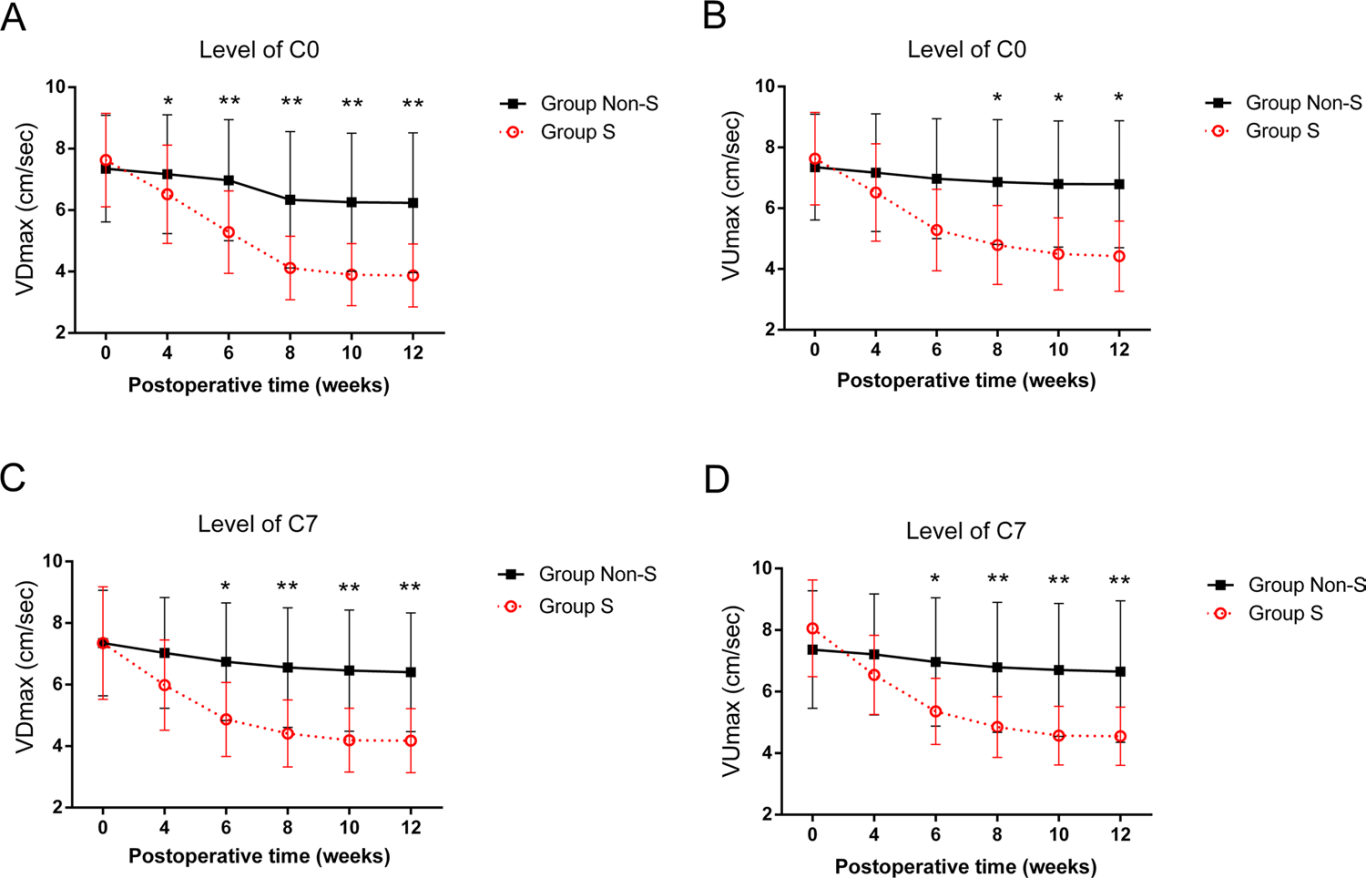
The changes of CSF flow at consecutive phases after kaolin-induction (group S vs group Non-S). At C0 level, after kaolin-induction, the peak velocities of CSF flow (VDmax and Vumax) were decreased from week 4 to 12 (**A,B**). At C7 level with a similar trend as at C0, after kaolin-induction, the peak velocities of CSF flow (VDmax and Vumax) were also decreased from week 4 to 12 (**C,D**). ^*^P < 0.05, ^**^P < 0.01.

From the comparisons between groups S and Non-S, the differences of the velocities of CSF flow at the foramen magnum (C0) and C7 levels were increased more obviously from week 4 to 12 after kaolin-induction, especially at postoperative week 6. The hydrodynamic changes of CSF flow at C0 and C7 levels were similar. At C0 level, to postoperative 12 weeks, the peak velocities of CSF flow (Vmax), including up-flow (VUmax) and down-flow (VDmax) of group S were much slower than group Non-S (VDmax: 3.9 ± 1.0 cm/sec vs 6.2 ± 2.3 cm/sec, *P* = 0.002; VUmax: 4.4 ± 1.2 cm/sec vs 6.8 ± 2.1 cm/sec, *P* = 0.011). Meanwhile, at C7 level, similar but more obvious differences were detected through the comparison between groups S and Non-S (VDmax: 4.2 ± 1.0 cm/sec vs 6.4 ± 1.9 cm/sec, *P* = 0.004; VUmax: 4.6 ± 0.9 cm/sec vs 6.7 ± 2.3 cm/sec, *P* = 0.003) (Fig. 3).

#### The relationship between scoliosis and CSF flow changes

**Figure 4:**
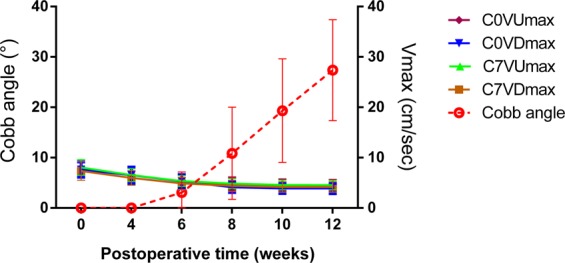
In group S, from preoperation to postoperative 4, 6, 8, 10, 12 weeks, the peak velocities (VDmax and VUmax) of CSF flow at C0 and C7 were decreased gradually, which was combined with the occurrence and progression of scoliosis from postoperative 6 weeks.

In the group S, after kaolin-induction, the VDmax and VUmax of CSF flow at the levels of C0 and C7 were diseased gradually. Especially, at week 12, being compared with the preoperative state, the VDmaxs at the level of C0 and C7 were decreased to 3.9 ± 1.0 cm/sec (*P* ≤ 0.001) and 4.2 ± 1.0 cm/sec (*P* ≤ 0.001) obviously, and the VUmaxs were also decreased to 4.4 ± 1.2 cm/sec (*P* ≤ 0.001) and 4.6 ± 0.9 cm/sec (*P* ≤ 0.001). Meanwhile, the spinal curve was observed initially from week 6, then the cobb angle of scoliosis was increased to 27.4 ± 10° at week 12 (Fig. 4).

**Figure 5:**
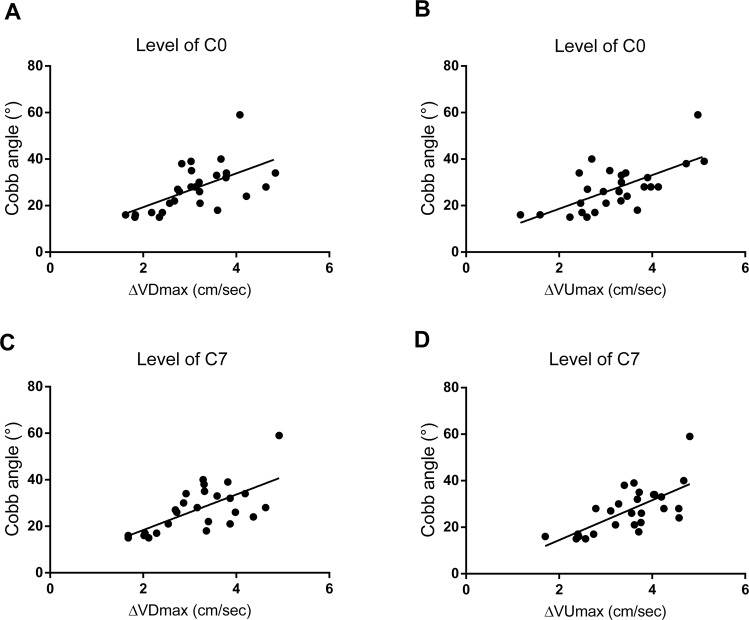
Relationship of CSF flow change and scoliosis formation at postoperative 12 weeks. ∆VDmax and Cobb angle at level of C0. Pearson correlation analysis shows a significant positive correlation of ∆VDmax versus Cobb angle (Pearson r = 0.595; *P* ≤ 0.001, n = 27). *Line* represents linear regression of data (y = 7.265x+ 4.808; r^2^ = 0.3542) (**A**). ∆VUmax and Cobb angle at level of C0. Pearson correlation analysis shows a significant positive correlation of ∆VUmax versus Cobb angle (Pearson r = 0.673; *P* ≤ 0.001, n = 27). Line represents linear regression of data (y = 7.240x+ 4.140; r^2^ = 0.4523) (**B**). ∆VDmax and Cobb angle at level of C7. Pearson correlation analysis shows a significant positive correlation of ∆VDmax versus Cobb angle (Pearson r = 0.668; *P* ≤ 0.001, n = 27). *Line* represents linear regression of data (y = 7.636x+ 3.103; r^2^ = 0.4460) (**C**). ∆VUmax and Cobb angle at level of C7. Pearson correlation analysis shows a significant positive correlation of ∆VUmax versus Cobb angle (Pearson r = 0.683; *P* ≤ 0.001, n = 27). *Line* represents linear regression of data (y = 8.588x+2.716; r^2^ = 0.4662) (**D**).

Moreover, in order to analyze the relationship between CSF flow changes and scoliosis formation, a correlational analysis was performed for all rabbits in group S at postoperative 12 weeks, and the results indicated a remarkable correlation of the final Cobb angle of scoliosis and the ∆VDmax (the decrease of VDmax from preoperative state to postoperative 12 weeks) and ∆VUmax (the decrease of VUmax from preoperative status to postoperative 12 weeks). At C0 and C7 levels, both ∆VDmax and ∆VUmax were positively correlated to the final scoliotic Cobb angle (*P* < *0.01*) (Fig. 5).

### Histological observation results

In the 25 rabbits for histological observation, 3 rabbits died: 2 was related to the mass blood loss in surgical process, 1 was to unclear reason.

**Figure 6:**
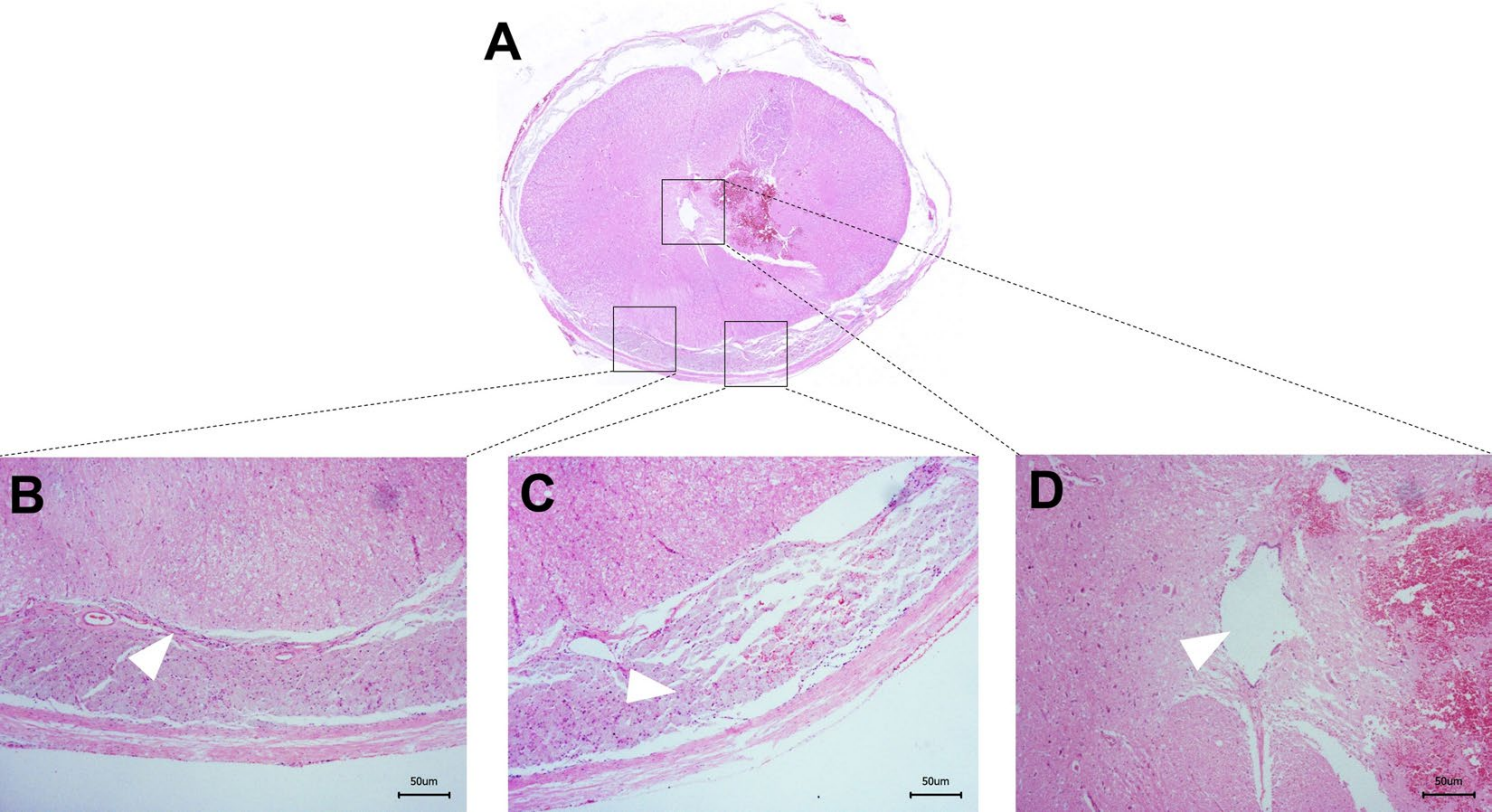
The extramedullary and intramedullary histological changes at postoperative 6-week with kaolin-induction, HE staining, 40× magnification (**A**). Focus of inflammatory infiltration in the thickening pia mater (arrow), 200× magnification (**B**). Focus of obstruction by the kaolin granulation and the inflammatory adhesion in the subarachnoid space (arrow), 200× magnification (**C**). Focus of the expanded central canal (arrow), 200× magnification (**D**).

**Figure 7:**
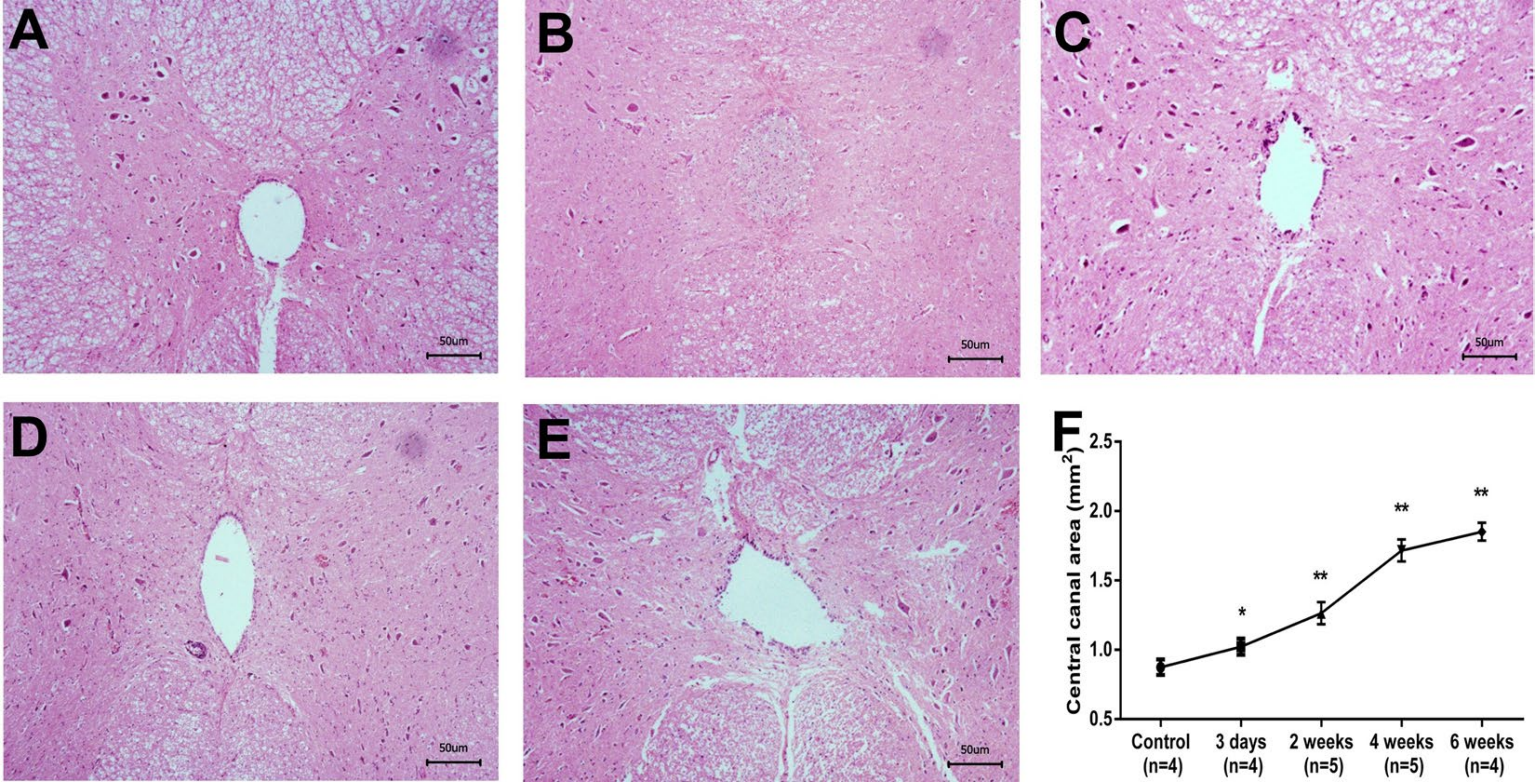
The changes of the central canal after kaolin-induction. To compare with the normal spinal cord tissue with regular and complete layer of ependymal cells (**A**), at postoperative 3-day with kaolin-induction, the central canal was tightly blocked by kaolin crystals, neutrophil cell and inflammatory exudation; the spinal cord parenchymal change was characterized by congestion, edema with neutrophil infiltration (**B**); at postoperative 2-week, the ependymal cells layer was interrupted and the central canal began to be expanded, and neutrophils infiltrated and macrophages engulfed the kaolin crystals in the spinal cord parenchyma with perivascular space (Virchow-Robin space) expansion (**C**); at postoperative 4-week, the central canal was further expanded with the ependymal cells proliferation, and lymphocytes infiltrated in the spinal cord parenchyma and accumulated in the expanded perivascular space (Virchow-Robin space) (**D**); at postoperative 6-week, the expansion of central canal became more obvious and asymmetric with disordered arrangement of surrounding structures (**E**). To compare with the control, the areas of the central canal gradually increased from postoperative 3-day to 6-week (**F**). *P < 0.05, **P < 0.01. (HE staining, 200× magnification).

Through the hematoxylin-eosin (HE) staining of spinal cord tissue being harvested at postoperative 3-day, 2-week, 4-week and 6-week after kaolin-induction, a series consecutive pathological changes of the spinal cord were observed, which was characterized by early inflammatory reaction, adhesion and blockage in the subarachnoid space and the central canal, perivascular space (Virchow-Robin space) enlargement, ependymal cell shedding, central canal expansion (Figs. 6 and 7). Moreover, in the postoperative 6 weeks, the area of central canal kept enlarging with time after kaolin-induction (Fig. 7).

## Discussion

Kaolin-induction is widely used to build SM animal model in different experimental researches. McLaurin and associates firstly described using kaolin injection into the cisterna magna consistently resulted in the development of chronic obstructive hydrocephalus in experimental animals^21^. Then a series of experimental SM models induced by kaolin injection into spinal cord and subarachnoid space have been reported^22–29^. Inflammatory reaction, apoptosis and secondary obstruction of the channels of CSF circulation are the main sources of using kaolin to induce SM models, which are also the important pathological basis of clinical SM occurrence. Especially, the formation of SM is critically related to the hypodynamic changes of CSF caused by several reasons.

In our early exploration of inducing SM by intramedullary injecting kaolin into spinal cord, we had ever noticed thoracic scoliosis occurrence in some experimental rabbits even without any spinal hydromyelia or syrinx formation^30^. After intramedullary kaolin injection, whether or not SM occurred, scoliosis would occur in a significant proportion of experimental animals. This was an interesting and meaningful phenomenon, which hinted that there seemed to be a connection between scoliosis and SM, but not a simple causal relationship. In order to further study the specific relationship between the change of CSF hydrodynamics and scoliosis formation, a modified kaolin injection process was used to induce experimental scoliosis in present study for improving the rate of scoliotic formation. After kaolin being injected into subarachnoid space and spinal cord layer by layer, at postoperative 6 weeks, spinal curve occurrence on the coronal plane was observed in 32.4% rabbits. To 12 weeks after kaolin-induction, the rate of scoliosis formation was up to 73.0%. Moreover, through MRI scanning, syrinx also started to be observed at postoperative week 6, which was mainly in the cervical-thoracic segments. Therefore, postoperative 6-week was an important phase, which represented the start point of scoliosis and syrinx after kaolin-induction. The period in the postoperative 6 weeks can be believed as the pre-scoliosis and pre-syrinx states, which would be very helpful in researches of this pathological process. Additionally, the final rate of syrinx formation was 70.3% at postoperative week 12. As an important part of pathological outcomes in the cord, syrinx observation outcomes by MR imaging was used to show there was no different of syrinx formation rates between the animals with and without scoliosis (74.1% vs. 70.0%, P > 0.05), which demonstrated there were similar influences to syrinx formation from kaolin-induced modeling approach no matter spinal coronal curve formation or not, which did not support there was a potential causal connection between scoliosis formation and SM.

In the previous clinical study, we had reported the use of electrocardiogram-synchronized cardiac-gated PC-cine MRI to study the differences between the patients of SM & CM-I malformation with and without scoliosis and found the worse dynamics of CSF flow in the patients with scoliosis^31^. For the scoliotic patients with syringomyelia, neurosurgical intervention such as posterior fossa decompression may prevent curve progression. The aim of neurosurgical decompression in these patients were to restore hydrodynamics of CSF flow, and the stop of scoliotic progression or even improved also hints there is some link between CSF hydrodynamics and scoliosis formation^32,33^. Recently, results from a study from Grimes *et al*., which was based on ptk7 mutant zebrafish with defects in ependymal cell cilia development and CSF flow, indicated the irregularities in CSF flow could be an underlying biological cause of idiopathic scoliosis^34^. In this study, through the comparison between the groups S and Non-S, the differences of the velocities of CSF flow became more obvious from postoperative week 4 to 12 after kaolin-induction, including at C0 and C7 levels, especially after postoperative 6 weeks. The hypodynamic changes of CSF flow at C0 and C7 levels were similar. In the group S, at the week 12, being compared with the preoperative state, the VDmax and VUmax at the level of C0 and C7 were decreased obviously. Meanwhile, the spinal curve was observed initially from the postoperative week 6 with obvious increase to the week 12. Moreover, from preoperation to postoperative 12 weeks, the changes of VDmax and VUmax positively correlated to the final scoliotic Cobb angle. Our results indicated there was a close relationship between the slow-down of CSF flow and scoliosis formation in the kaolin-induced rabbit model. The hypodynamic changes of CSF flow could play an important role in the process of scoliosis formation.

Moreover, to focus on the postoperative 6-week after kaolin-induction as an important period including pre-scoliosis and pre-syrinx states, consecutive histological observations were also performed in this study. The histological changes was characterized by early inflammatory reaction, adhesion and blockage in the subarachnoid space and the central canal, perivascular space (Virchow-Robin space) enlargement, ependymal cell shedding, central canal expansion, etc. Combining with the significant changes of CSF flow at 4–6 weeks after kaolin induction in the previous part of this study, the histological changes indicated that the hydrodynamic change to CSF flow could not only be derived from the subarachnoid inflammatory adhesion and blockage by kaolin crystal itself, but also from intramedullary lesions. The involved pathological processes could be speculated as follows: kaolin-induced subarachnoid inflammation caused the normal CSF flow to be blocked, which in turn caused increased subarachnoid pressure; meanwhile, the intramedullary inflammatory response could lead to the opening of the perivascular space as well as the CSF with increased pressure entering into the central canal through it. Additionally, the kaolin crystals and intramedullary inflammatory reaction also could cause the central canal to be blocked, which would increase the accumulation of CSF in the central canal and the final formation of syrinx. Therefore, this animal scoliotic modeling method intervened the normal CSF flow by multiple ways, especially the obstruction of the subarachnoid space and the central canal. Moreover, the following hypodynamic change of CSF flow is prior to the formation of scoliosis, which suppled a powerful evidence of the consecutive hypodynamic change of CSF flow could be an important potential cause of scoliosis formation.

There are some limitations of the current study. In this study, we could not find a reliable approach to quantitatively analyze whether there were histological differences in the cord and subarachnoid space between the animals with and without scoliosis. Although this study primarily established the correlation between hypodynamic change of CSF flow and scoliosis formation, the specific relationship between them was still obscure. The different degree of cord damage and arachnoiditis could result in lower CSF flow velocities and scoliosis development. In that case, the possibility of lower CSF flow velocities and scoliosis development would be two symptoms of a more severe cord damage and/or arachnoiditis, which could not be excluded in this study. The aim of this study was to introduce hypodynamic CSF flow as a possible factor of soloistic pathogenesis, more evidences and researches are needed to explain how the hydrodynamic change of CSF flow affects the structure of spine as well as the following scoliosis occurrence and progression. The process maybe through intervertebral discs, paravertebral muscles, intraspinal structures or other factors, which still needs further researches in future. And these researches would be helpful to reveal the potential mechanisms of scoliosis formation and to explore future novel treatments.

In summary, scoliosis can be induced in most of the rabbits by kaolin injection into cervical spinal cord and subarachnoid space. This kaolin-induced scoliosis model could be a good choice for the pathogenesis exploration of idiopathic scoliosis. In this animal model, postoperative 6-week is a start of scoliosis after kaolin-induction, and continuous hypodynamic change of CSF flow was correlated to the formation of scoliosis, which could be an important factor of scoliotic pathogenesis being explored furtherly.

## Materials and methods

### Animals

This study was carried out in strict accordance with the recommendations of the Guide for the Care and Use of Laboratory Animals of the National Institutes of Health, following ethical approval from the Animal Care and Ethics Committee of the Kunming Medical University (# SYDW20080125001). A total of 75 Japanese white rabbits (Hunan SJA Laboratory Animal Co., Ltd., Hunan, China) were included in this study, aged 2–3 months old and weighting 1.8–2.5 kg. Each animal was kept in an individual cage and had free access to food and water. The room temperature range was 20–28 °C, with a relative humidity of 35–60% and a 12 hours light-dark cycle. In the 75 rabbits, 25 were used for histological observation and 50 were for imaging observation (Table 2).

**Table 2:** Summary of Experimental Rabbits.

n	Injection	Purpose	Postop survival time
10	saline	IO	12 weeks
40	kaolin	IO	12 weeks
5	—	HO	—
5	kaolin	HO	3 days
5	kaolin	HO	2 weeks
5	kaolin	HO	4 weeks
5	kaolin	HO	6 weeks

*IO indicates imaging observation; HO indicates histological observation.

### Scoliosis induction

**Figure 8:**
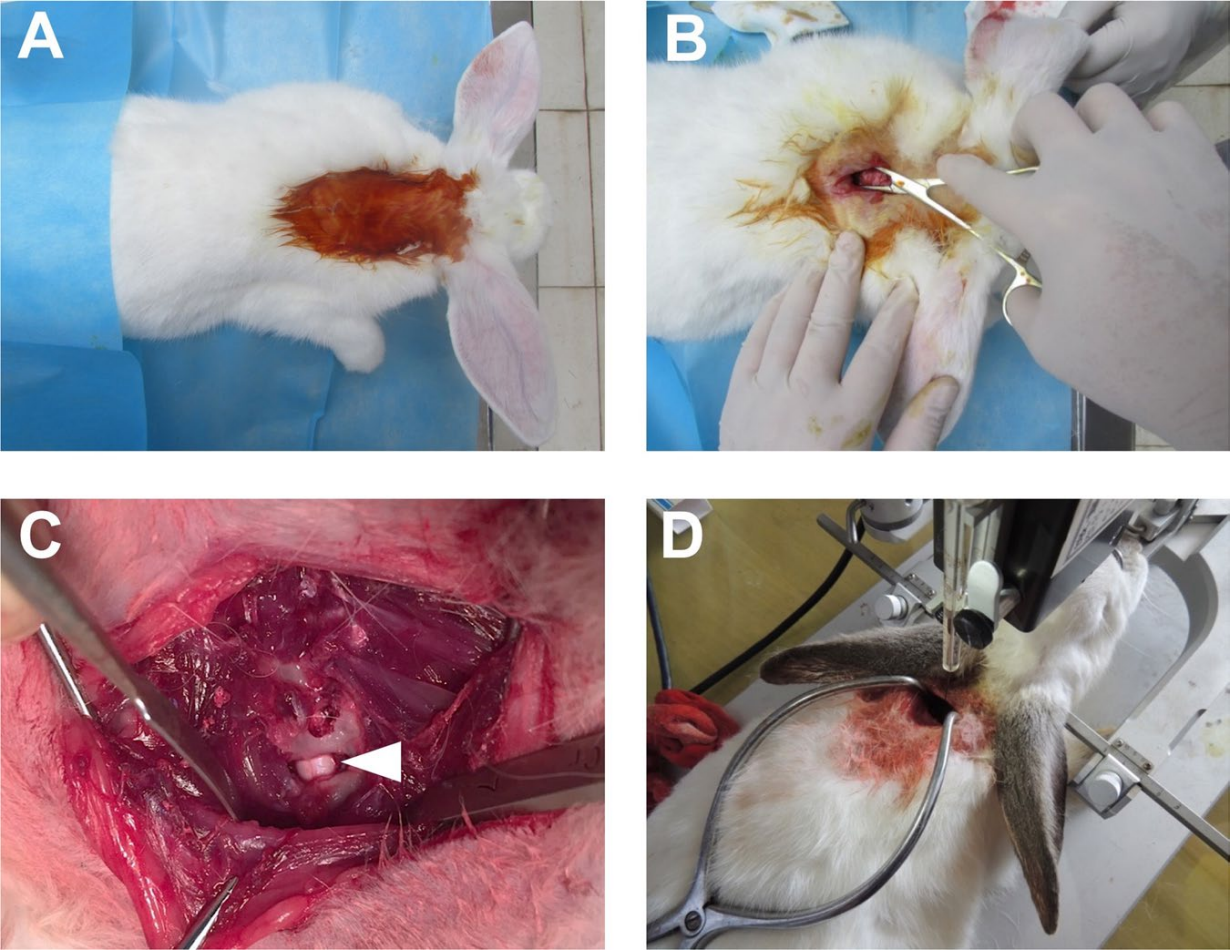
The process of building a rabbit scoliotic model by kaolin-induction. The experimental rabbit was placed prone on a self-made frame to raise the cervicothoracic junction (**A**). A 2 cm midline incision was made from the seventh cervical vertebra (C7) to the first thoracic vertebra (T1), and the basis of C7 spinous process was exposed (**B**). After cutting the C7 spinous process, a tiny part of the lamina and ligamentumflavum were resected for exposing the dura (arrow) (**C**). Kaolin was injected from the subarachnoid space to the center of the spinal cord layer by layer through the dura being navigated by a stereotaxic apparatus (**D**).

Before surgery, through the ear marginal vein, the general anesthesia was induced by intravenous administration of 3% pentobarbital sodium at a dose of 30 mg/kg (Siagma, America). The skin was shaved and prepared with povidone iodine. Then animals were placed prone on a self-made U-shape frame to raise the cervicothoracic junction for avoiding the interrupt from the scapula to facilitate next steps. A posterior median longitudinal incision (about 2 cm length) was made from C7 to the first thoracic vertebra (T1). Then the C7 spinous process was cut off, and at its basis, a tiny part of the lamina and ligamentum flavum were resected for exposing the dura. An 100 microliter glass microinjector with a 28-gauge needle (Qing Niu Medical Apparatus and Instruments Factory, China) was held by a stereotaxic apparatus (SR-6N, Narishige Group, Japan) to be used for the dura puncture. The site of puncture was near the middle line of the dura but avoiding the dorsal vessels, the depth was 1.0–2.0 mm under the dura. Then 0.1 ml of 25% kaolin (Sigma, America) was injected from the subarachnoid space to the center of the spinal cord layer by layer (Fig. 8). Wounds were closed with multilayers silk sutures. After the surgery, all the rabbits were fed under close observation. The kaolin-induction processes were performed in 60 rabbits randomly: 40 were used for imaging detections and 20 were for histological observation (Table 2).

### The sham animal

Another 10 rabbits were selected randomly as the sham: same surgical process was performed but using the 0.9% saline to instead of the 25% kaolin.

### Imaging detection

In order to study the relationship between scoliosis formation and hydrodynamic changes of CSF flow, 40 kaolin-inducted rabbits were randomly selected for consecutive PC-cine MRI detections at consecutive phases. Meanwhile, radiological detections were performed in the 40 rabbits and the 10 sham rabbits for observing the formation of scoliosis.

#### Radiological observation

In a prone position, the full-length posterior-anterior radiograph of the spine was taken with head and body being straightened at postoperative 4, 6, 8, 10, 12 weeks under the general anesthesia. The Cobb method was used to measure the curve on the coronal plane in order to record the progression of scoliosis.

#### PC-cine MRI detection

The methodology of PC-cine MRI observation was based on our previous study^31^, and the MRI scanning condition and parameters were adjusted to be suitable for rabbit detection. The MRI examinations were performed with a 1.5-T MRI scanner (Sonata, Siemens, Germany) using cervical and spinal coils. PC-cine MRI scans were performed preoperatively and 4, 6, 8, 10, 12 weeks postoperatively.

Ten minutes before MRI scanning, the general anesthesia was induced by intramuscular injection of 3% pentobarbital sodium (Siagma, USA) through the gluteus maximus at a dose of 30 mg/kg. MRI scanning was started at a prone position with steady heart rate and respiration. Sagittal and axial T1-weighted, T2-weighted imaging sequences of the brain and cervical spinal cord were collected. Synchronized cardiac gating was used to divide the cardiac cycle into 23 images. CSF flow studies were performed at the levels of C0 and C7 in the axial plane with following sequence: TR 1800 msec/TE 93 msec, flip angle 180°, slice thickness 4 mm, matrix 256 × 196, and encoding velocity 20 cm/sec.

**Figure 9:**
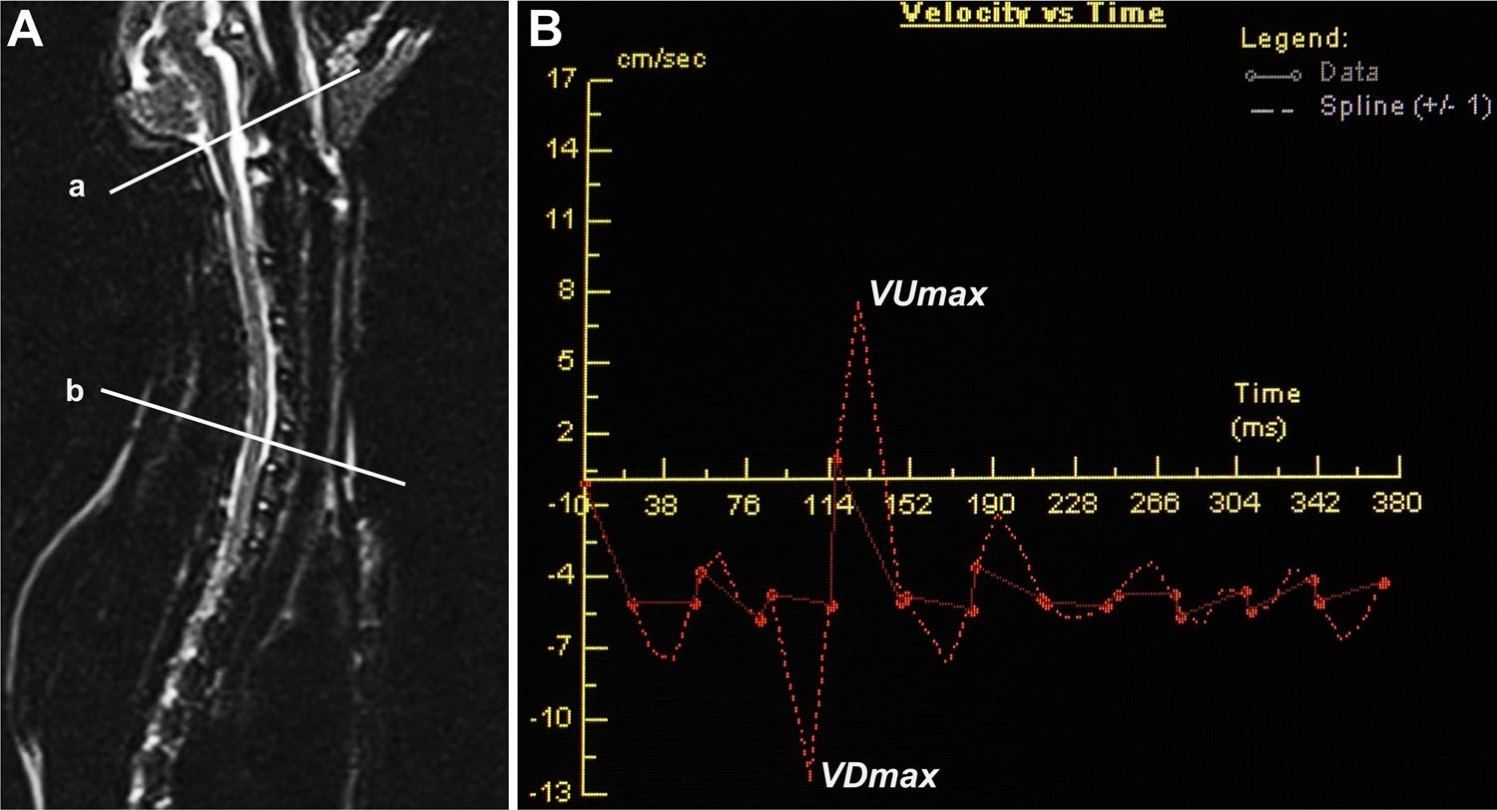
The measurement of CSF flow by PC-cine MRI observation. CSF flow was measured at C0 (line a) and C7 (line b) levels (**A**). CSF flow was analyzed by Argus postprocessing program, the peak velocities of CSF flow were measured on the basis of the CSF velocity-time curve, including up-flow (marked by VUmax) and down-flow (marked by VDmax) (**B**).

The acquired phase-contrast images were transferred to Argus software (Siemens, Germany) for CSF flow analysis. Regions of interest (ROIs) were selected in the axial plane: 4 ROIs were evenly placed in the anterior and posterior subarachnoid spaces at C0 and C7 levels. The peak velocities of CSF flow, including VUmax and VDmax were measured and averaging values at the 4 different ROIs were recorded at different phases (Fig. 9).

#### Animal grouping

According to the occurrence of scoliosis at the end of week 12 after kaolin-induction, the kaolin-induced rabbits were divided into two groups: scoliosis (group S) and non-scoliosis groups (group Non-S). On the basis of the grouping, the changes of CSF flow velocity were reviewed and analyzed being combined with/without scoliosis formation: 1. what was the role of the CSF flow velocity in curve formation? 2. what the relationship between the change of CSF flow velocity and the curve progression?

### Histological observation

From our previous study about the kaolin-induced syringomyelia-associated scoliosis rabbit model, postoperative week 6 were the common initial points of syrinx and scoliosis being detected, which included important information about the occurrence of this pathological process^30^. Therefore, in order to study structural changes in the spinal cord after kaolin induction, the rest 20 kaolin-inducted rabbits and 5 normal ones were sacrificed at different phases in postoperative 6 weeks, including postoperative 3-day, 2-week, 4-week and 6-week (Table 2). Rabbits were anesthetized with 3% pentobarbital sodium (30 mg/kg, Siagma, USA) and transcardially perfused with saline, followed by 4% paraformaldehyde. Then the spinal cords were carefully dissected, labeled as cephalic/caudal ends and fixed in 4% paraformaldehyde at 4 °C for 24 h. A 3-cm length of spinal cord segment, centered on the kaolin injection site, was excised from the whole spinal cord and embedded in paraffin. All harvested spinal cord segments were sliced transversely into 5-μm-thick sections and HE staining were used for evaluation of structural changes. Meanwhile, the border of the central canal was outlined by Image-Pro Plus 6.0 (Media Cybernetics, Inc., Rockville, MD, USA) software to measure and calculate its area.

### Statistical analysis

All numerical data are presented as expressed as mean ± standard error (S.E.M). Independent sample t test was used to test the differences of the velocities of CSF flow between groups S and Non-S, and the changes of the central canal area at postoperative different phases. Correlation studies were done using Pearson Correlation test when appropriate. Differences between two groups were assessed by independent *t* test, and categorical variables were compared using Chi-square test. Statistically significant differences were accepted at *P* < *0.05*. Statistical chart was designed with GraphPad Prism 6.0 (San Diego, CA, USA).

### Compliance with Ethical standards

The experimental protocol for this study was approved by the Animal Care and Ethics Committee of the Kunming Medical University (# SYDW20080125001). The approval was obtained before the start of the experiments. A licensed breeder had bred all animals for the sole purpose of being used in animal experiments. All efforts were made to minimalize animal suffering.

## Data Availability

All data generated or analysed during this study are included in this published article and are available from the corresponding author on reasonable request.
